# OA08.01. Cholecystokinin antagonizes opioid function during electroacupuncture inhibitory effect on pressor reflex in rats

**DOI:** 10.1186/1472-6882-12-S1-O29

**Published:** 2012-06-12

**Authors:** M Li, S Tjen-A-Looi, Z Xu, E Choi, J Ho, J Longhurst

**Affiliations:** 1 University of California, Irvine, Department of Medicine, Irvine, USA

## Purpose

Acupuncture or electroacupuncture (EA) is increasingly being accepted as a viable therapy in the United States and may serve as an alternative treatment to drug therapy in patients with mild to moderate hypertension. We have demonstrated that EA acts through an opioid mechanism in the rostral ventrolateral medulla (rVLM) to inhibit sympathoexcitatory reflexes induced by gastric distention. Cholecystokinin octapeptide (CCK-8), a gastrointestinal peptide hormone, is present throughout the central nervous system and mediates pain and anxiety. CCK-8 interacts with CCK1 and CCK2 receptors. The present study investigated the hypothesis that CCK-8 in the rVLM limits the modulatory action of EA effect on sympathoexcitatory responses.

## Methods

Experiments were performed on male rats anesthetized with ketamine and α-chloralose who were subjected to repeated gastric distension every 10 minutes.

## Results

EA (2Hz, 0.5ms, 1-4mA) at Jianshi-Neiguan (P5-P6) acupoints applied for 30 min followed by microinjection of CCK-8 (0.15mM, 50 nl) into the rVLM in 7 rats reversed the EA modulatory response from 11.0 (± 2.0) to 19.1 (± 2.8) mmHg. Saline injections into the rVLM at the end of 30 min of EA did not influence the prolonged inhibition of reflex elevations in blood pressure in five other animals. Alternatively, devazepide (0.5 mM, 50 nl), a CCK1 antagonist microinjected into the rVLM of six rats that initially did not respond to 30 min of EA, led to conversion during a second application of EA. Microinjection of the vehicle of devazepide (50 nl) into rVLM did not modify the nonresponsiveness to EA application (n=2).

## Conclusion

These data suggest that CCK-8 through its action on CCK1 receptors limits the action of EA in the rVLM of rats in modulating reflex hypertension. (Supported by AHA 10POST4190125, NIH HL-63313 and HL-072125).

